# Tinnitus-Related Changes in the Inferior Colliculus

**DOI:** 10.3389/fneur.2015.00061

**Published:** 2015-03-30

**Authors:** Joel I. Berger, Ben Coomber

**Affiliations:** ^1^Medical Research Council Institute of Hearing Research, University of Nottingham, Nottingham, UK

**Keywords:** acoustic over-exposure, salicylate, auditory, inferior colliculus, tinnitus, midbrain

## Abstract

Tinnitus is highly complex, diverse, and difficult to treat, in part due to the fact that the underlying causes and mechanisms remain elusive. Tinnitus is generated within the auditory brain; however, consolidating our understanding of tinnitus pathophysiology is difficult due to the diversity of reported effects and the variety of implicated brain nuclei. Here, we focus on the inferior colliculus (IC), a midbrain structure that integrates the vast majority of ascending auditory information and projects via the thalamus to the auditory cortex. The IC is also a point of convergence for corticofugal input and input originating outside the auditory pathway. We review the evidence, from both studies with human subjects and from animal models, for the contribution the IC makes to tinnitus. Changes in the IC, caused by either noise exposure or drug administration, involve fundamental, heterogeneous alterations in the balance of excitation and inhibition. However, differences between hearing loss-induced pathology and tinnitus-related pathology are not well understood. Moreover, variability in tinnitus induction methodology has a significant impact on subsequent neural and behavioral changes, which could explain some of the seemingly contradictory data. Nonetheless, the IC is likely involved in the generation and persistence of tinnitus perception.

## Introduction

The most common etiology of chronic tinnitus in the human population arises from exposure to excessive levels of noise (1). Tinnitus is suggested to affect between 8 and 15% of the population and is extremely debilitating in ~1% (2). It is often perceived as a ringing sound (the word tinnitus originates from the Latin *tinnire*, which translates as “to ring”), but characteristics vary across individuals (3). While the cause of chronic tinnitus was believed to reside within the inner ear (4), it is now widely accepted that changes in the central auditory system are pivotal in generating the phantom percept, as symptoms persist following cochlea ablation (5) or severance of the auditory nerve (AN) (6). However, in the early stages of tinnitus development, central changes still appear to be dependent upon peripheral activity (7, 8).

The inferior colliculus (IC) is a near-obligatory relay in the ascending auditory pathway, a point at which virtually all lemniscal and extra-lemniscal ascending inputs converge (9). As such, pathophysiological changes in the IC can alter all aspects of auditory perception. Thus, the IC has been putatively proposed as an important structure in central mechanisms that underlie subjective tinnitus, and has been widely studied in this context (10).

In this review, we concentrate on research focused on the role of the IC in tinnitus. This translates – in the main – to animal models of tinnitus, which have been extensively used to identify putative neural correlates of tinnitus in the midbrain, such as changes in patterns of neural activity and alterations in neurotransmission. While acoustic over-exposure (AOE), or damage caused by intense sound, is the most prevalent cause of tinnitus in humans, here we also consider animal studies examining the effects of pharmacologically induced tinnitus in an attempt to consolidate the nuanced similarities and differences between models. In addition, we consider data derived from imaging studies with tinnitus patients. Finally, we discuss IC pathophysiology as a contributing factor in the context of different tinnitus models, and the likelihood of tinnitus generation occurring through interactions with other neural circuitries, such as limbic and somatosensory systems.

## Changes in the IC Following AOE

Short-term changes following noise exposure are summarized in Table 1. In the *immediate* aftermath of intense noise exposure, a number of studies have demonstrated altered patterns of neural activity in the IC. In mouse brain slice recordings spontaneous firing rates (SFRs) of IC neurons decreased (11). Furthermore, auditory-evoked responses in the IC of awake guinea pigs were reduced immediately after noise exposure (12, 13). In contrast, other groups have found no immediate change in IC firing rates recorded *in vitro* (14), or increased neuronal firing *in vivo* 12 h post-AOE (15). Increased neural activity in the IC was implied by elevated c-Fos (a gene associated with neuronal depolarization) in rats (16). The most likely explanation for immediate decreases in neural activity in the IC is the well-documented decrease in cochlear and AN output (17–20). On the other hand, elevated activity in the IC might reflect rapid plastic changes to compensate for diminished input to the IC, such as a suppression of lateral inhibition (21). The spectrum of changes caused by AOE probably arises from variations in exposure duration/sound level, differences in measurement time-points following AOE, or possibly species differences.

**Table 1:** **Pathophysiology in the IC associated with the short-term effects of acoustic over-exposure**.

	+	−
**NEURAL ACTIVITY**		
Spontaneous activity	Mulders and Robertson (15) and Wang et al. (22)	Basta and Ernst (11)
Auditory-evoked activity	Wang et al. (22)	Popelar et al. (12) and Sun et al. (13)
**NEUROTRANSMISSION**		
Excitation
NMDA		Dong et al. (23)
Inhibition
GABA	Tan et al. (24)	Dong et al. (23) and Szczepaniak and Moller (25)
GAD_65_		Milbrandt et al. (26)
GAD_67_	Abbott et al. (27)	Dong et al. (23)
GlyR		Dong et al. (23)
**GENE EXPRESSION**		
c-FOS	Saint Marie et al. (16)	
BDNF	Meltser and Canlon (28) and Tan et al. (24)	
MAPK	Meltser and Canlon (28)	

*Elevations or enhancements in signaling are indicated by “+” and reductions by “−*.”

Subsequently, within 2 weeks post-AOE, there is general and widespread agreement that SFRs in the IC increase (14, 22, 23, 29, 30). This hyperactivity is often restricted to regions that respond preferentially either to the exposure frequency or frequencies above, but recent evidence suggests that this is not always the case (31, 32). Long-term changes following noise exposure are summarized in Table 2.

**Table 2:** **Pathophysiology in the IC associated with the long-term effects of acoustic over-exposure**.

	+	−
**NEURAL ACTIVITY**		
Spontaneous activity
Electrophysiology	Coomber et al. (31), Dong et al. (23), Groschel et al. (14), Manzoor et al. (29, 33), Mulders and Robertson (7, 8), Ropp et al.(32), Vogler et al. (30), and Wang et al. (22)	
Metabolic markers	Holt et al. (34)	
Burst firing and synchrony	Bauer et al. (35) and Coomber et al. (31)	
Auditory-evoked activity	Berger et al. (36) (altered response profiles), Izquierdo et al. (37) (tonotopic reorganization), and Wang et al. (20, 21)	
**NEUROTRANSMISSION**		
Excitation
Glutamate	Godfrey et al. (38)	
Inhibition
GABA	Godfrey et al. (38)	
GABA_A_	Milbrandt et al. (26)	Dong et al. (23, 39)
GAD_65_		Abbott et al. (27)
GAD_67_		Abbott et al. (27)

*Elevations or enhancements in signaling are indicated by “+” and reductions by “−*.”

### Changes in neurotransmission following acoustic trauma

Early work examining γ-aminobutyric acid (GABA)-mediated neural signaling indicated decreased GABAergic inhibition in the central nucleus of the inferior colliculus (CNIC) following tonal noise exposure (25). Thus, a potential mechanism by which chronic, AOE-induced hyper-excitability could be mediated in the midbrain was identified. GABAergic inhibition in the IC is widespread, shapes acoustically evoked non-monotonicity, offset inhibition and binaural inhibition [for a review, see Ref. (40)], and synaptic responses in rat brain slices (41).

There is a reasonable body of evidence suggesting that inhibition in the IC is altered rapidly following AOE, although the time scales vary considerably between studies. Furthermore, in the longer term, inhibition is altered permanently, affecting the balance of inhibition and excitation.

Levels of glutamic acid decarboxylase (GAD), the main enzyme responsible for GABA production, are altered following AOE. Immediately after noise exposure, the GAD_67_ isoform, which is widespread within neurons and produces GABA for wide-ranging functions (42, 43), was elevated in terms of protein levels and the density of stained cells (27). After 30 days, GAD_67_ protein levels were *reduced* relative to unexposed controls, but with no significant changes in stained cell densities. At the same time point, protein levels of GAD_65_ (localized to synaptic terminals and responsible for transiently synthesizing GABA for fast neurotransmission) were also reduced, but to a lesser extent.

Suneja et al. (44) found that GABA release in CNIC was immediately augmented by both ossicle removal and cochlear ablation, although in their study GABA release remained elevated. Meanwhile GABA uptake was depressed, providing further evidence for pathologically altered inhibition in the IC, in this case caused by invasive peripheral trauma.

In contrast, Milbrandt et al. (26) observed a significant decrease in GAD_65_ in rats in the short-term when using a noise exposure paradigm specifically designed to target high frequencies, but this recovered to near-normal levels when examined 30 days after AOE. Furthermore, GAD_65_ recovery at 30 days coincided with a significant increase in [^3^H]muscimol binding, indicative of increased GABA_A_ receptor binding sites. Dong et al. (23) also found immediate reductions in gene expression related to inhibition after noise exposure, but this was also the case for genes related to excitation, despite the absence of a change in spontaneous firing. In both cases, expression generally returned to near-normal levels over time. Moreover, GABA_A_α1 expression, a receptor subunit involved in fast inhibitory neurotransmission, decreased in tonotopically organized regions of the IC that responded to frequencies close to the exposure frequency (39).

Rats subjected to a cochleotomy, on the other hand, displayed more consistent reductions in GAD_67_ expression over time (45). In the same study, decreased expression of the α1 subunit of glycine receptors was also observed under certain conditions, yet there were no changes in other glycine receptor subunits, or in a variety of components that make up GABA_A_ or *N*-methyl-d-aspartate (NMDA) receptors. Interestingly, Argence et al. (46) were subsequently able to reverse the down-regulation of GAD_67_ and GlyRα1 by electrically stimulating the deafferented AN. This effect proved to be temporary, disappearing within 5 days after stimulation ceased. However, it should be noted that the breadth of changes induced by a complete cochleotomy are likely to differ significantly from a more selective approach, such as AOE.

In hamsters, moderate yet significant elevations of both glutamate and GABA were observed in the IC after bilateral AOE, yet no change was seen in glycinergic signaling, or in levels of neurotransmitters in other parts of the auditory system, including the cochlear nucleus, medial geniculate body (MGB), and auditory cortex (38). These changes were concurrent with elevations in aspartate (relating to glutamate synthesis) and decreased levels of taurine (which can be linked with GABA or glycine function). Of particular interest was the time-span over which a presumed shift in the balance of inhibition and excitation occurred; in this study, hamsters were examined 5 months after AOE and compared with unexposed controls. In an earlier paper, Tan et al. (24) also demonstrated an increase in GABA-positive neurons, combined with elevated brain-derived neurotrophic factor (or BDNF) 6 days following AOE. Contrastingly, using an imaging technique to quantify GABA and glutamate, Brozoski et al. (47) demonstrated no excitatory or inhibitory changes in the IC, but significant changes in the dorsal cochlear nucleus (DCN), MGB, and primary auditory cortex (AI) of normal-hearing rats with tinnitus (induced by unilateral AOE and confirmed behaviorally).

### Chronic hyperactivity in the IC

Changes in inhibitory neurotransmission, such as those described above, could result in the unmasking of previously dormant inputs within these regions. Such a mechanism might feasibly contribute to maintaining hyperactivity induced by AOE, as well as contributing to tonotopic reorganization in the IC, which in some instances has been found to occur (22, 37). However, the origin of increased spontaneous activity in the IC, in terms of generation and persistence, and whether it depends upon intrinsic processes or external input, has only been explored more recently.

Manzoor et al. (33) demonstrated that IC hyperactivity was significantly reduced by ablation of the DCN 2–3 weeks following AOE, suggesting that an increase in SFRs at the level of the midbrain occurred as a result of increased activity extrinsic to this structure. This suggests that IC hyperactivity, at least in the early stages following AOE, is not a result of intrinsic plasticity. In support of this, Brozoski et al. (48) demonstrated that bilateral DCN lesions prior to AOE prevented the development of behavioral evidence of tinnitus, although this was not the case for unilateral lesions.

However, these results do not rule out the possibility that IC hyperactivity becomes an intrinsic process over a period of time, independent of ascending input. Bilateral DCN ablation 3–5 months after AOE did not abolish behavioral evidence of tinnitus (49), while cochlear ablation only modulated IC hyperactivity within 6 weeks of AOE (7, 8). In light of the current evidence, IC hyperactivity likely depends on ascending input in the early stages post-AOE, but subsequently becomes self-sustaining.

Interestingly, recent findings by Ropp et al. (32) suggest that the ventral cochlear nucleus (VCN) may play a substantial role in IC hyperactivity; recording 1–4 months after AOE, IC neurons receiving input from VCN (identified by electrophysiological response profiles) were hyperactive, while IC neurons receiving input from DCN were not. Thus, at a later stage, IC hyperactivity appears to be independent of input from DCN, but the VCN may be implicated in the persistence of increased SFRs. However, more research examining how VCN modulates IC firing following AOE is necessary to draw definite conclusions.

## Linking Neural Pathophysiology to Tinnitus Perception

Some of the studies outlined above highlight a series of consequences of hearing loss that could underlie tinnitus generation. However, the implementation of a complementary behavioral test in animals is an essential step in correlating neural changes with evidence of tinnitus perception. This enables delineation of changes that might relate to tinnitus from those that occur as a by-product of hearing loss. This is a pertinent point, as hearing loss does not invariably lead to the generation of tinnitus (50). Early behavioral tests relied on extensive training to produce a conditioned response (51–53), although more recent studies have exploited unconscious reflexes to determine tinnitus-like behavior, often known as the gap prepulse inhibition of acoustic startle (GPIAS) test (54–58). While the efficacy of these behavioral tests for detecting tinnitus *per se* has recently been questioned (59, 60), an effective, objective test is vital if we are to be confident in attributing neural changes to tinnitus rather than to hearing loss.

Behavioral evidence of chronic tinnitus emerges at a period beginning five weeks after AOE (61). Around the same time, hyperactivity is evident in the IC (7, 8). However, two recent studies have suggested that this hyperactivity may not be sufficient as a sole generator of tinnitus. In AOE-treated guinea pigs and rats, increased spontaneous neural activity was present even in the absence of behavioral evidence of tinnitus (31, 32), although these findings may be influenced by interpretation of behavioral data. Given that the majority of studies demonstrate that IC hyperactivity is evident following noise trauma, but can also be present in the absence of tinnitus, this suggests that increased SFRs, at least at the level of the IC, may be necessary but not sufficient to explain tinnitus generation.

## Other Changes Occurring within the IC

In addition to elevated spontaneous activity, a number of other changes in neuronal firing properties induced by noise exposure have been linked to tinnitus, such as increased burst firing in chinchillas (35) and guinea pigs (31). Burst firing patterns have previously been associated with neural synchrony (62); that is, correlated firing across a population of neurons. Increased neural synchrony has also been directly measured by correlating firing patterns across different IC neurons (35). It has been suggested that neural synchrony underlies auditory perception [for a review, see Ref. (63)] so it is entirely plausible that synchronous activity in the absence of auditory input could manifest as a tinnitus percept. Indeed, some treatment options focus solely on disrupting neural synchrony [see Ref. (64)]. However, the importance of synchrony in the IC to tinnitus generation and maintenance is yet to be elucidated.

The uncertainty surrounding the role of the IC in tinnitus generation can in part be attributed to differences between the three major divisions: the CNIC, the external nucleus of the inferior colliculus (ICx), and the dorsal cortex (ICd). The responses of these divisions are relatively distinct, with different proposed functional roles (65). It is therefore reasonable to assume that responses to reduced input following AOE might also differ. Although IC sub-divisions can be identified physiologically or histologically (66, 67), the delineations and borders are often not clear and thus many studies do not explicitly state which area was examined. Interestingly, using manganese-enhanced magnetic resonance imaging (MRI) in rats to examine a variety of auditory and non-auditory nuclei, 2 days post-AOE, Holt et al. (34) found that the ICd was the only area that exhibited consistent increases in activity following two different tinnitus inducers. ICd is the predominant site of corticocollicular descending input (68–72), although there is some overlap near the border with CNIC where ascending connections are also present (68, 73). Furthermore, there are well-defined intrinsic connections between ICd and CNIC [for a review, see Ref. (74)]. Nonetheless, these data could implicate the descending forebrain in altering spontaneous activity in the IC (75). Indeed, when focal electrical stimulation was applied to the auditory cortex, thus activating corticofugal pathways, this caused temporary shifts in IC frequency representation (76). Thus, tonotopic restructuring in the IC following acoustic trauma (22, 37) could feasibly be mediated, at least in part, by descending input.

Within the CNIC, AOE-induced changes were not restricted to particular response profiles (30). We recently demonstrated that the proportional balance of response profiles can be altered by AOE; the proportion of onset-type responses increased significantly, while the proportion of single-units with sustained firing patterns decreased (36). However, the presence or absence of behavioral tinnitus had no bearing on the balance of onset and sustained profiles; in other words, this effect likely reflected long-term changes induced by AOE.

A potential confound to any study examining changes following AOE pertains to the exposure paradigm itself; that is, different sound levels, durations, and frequencies of noise exposure could result in diverse neural changes. Suggestive of this, hamsters were more likely to develop behavioral evidence of tinnitus when subjected to an increased duration of an otherwise identical noise exposure (52). Moreover, Meltser and Canlon (28) found that an AOE paradigm designed to cause “permanent” damage resulted in transient activation of BDNF and a variety of mitogen-activated protein kinases, whereas temporary damage was only associated with activation of selected p38 kinases. They reasoned that these effects were indicative of plastic changes resulting from reduced sensory input, dependent on the magnitude of insult. AOE paradigms vary substantially between studies, while species differences in susceptibility to AOE-induced damage (77) prevent implementation of a standardized protocol. Accordingly, disparity in AOE protocols provides a possible explanation for seemingly conflicting results.

The short-term and long-term effects of AOE, specifically, are summarized in Tables 1 and 2. To conclude, the short-term effects could be described as: (1) immediate changes in spontaneous and auditory-evoked neural activity, which are variable perhaps due to experimental protocol, (2) predominantly, reductions in mediators of both excitation *and* inhibition, with a couple of exceptions, and (3) elevated immediate-early gene expression, indicative of altered patterns of neuronal activity. In terms of long-term changes after AOE, studies indicate, overwhelmingly, that (1) spontaneous, synchronous, and auditory-evoked activity are elevated, and (2) changes in components of inhibition in particular are complex, but probably underlie an overall change in the balance of excitation and inhibition.

## IC Pathology in Models of Pharmacologically Induced Tinnitus

Sodium salicylate, an analog of acetylsalicylic acid (the active ingredient in aspirin), is ototoxic at high doses and induces transient tinnitus in humans (78). Salicylate is used experimentally to induce tinnitus in animal models (53, 54, 79–81). A significant benefit in using salicylate as a tinnitus-inducing agent, is that – compared with AOE – the behavioral effects are largely homogeneous. Thus, one can reliably predict that nearly all of the animals will exhibit behavioral evidence of tinnitus.

The definitive mechanisms by which salicylate causes tinnitus are unknown, although neural activity is affected at multiple levels of the auditory pathway. This includes peripheral effects, such as altered outer hair cell electromotility (82), as well as central effects, from AN fibers through to auditory cortex (83–85). Early work indicated that – in cats at least – secondary auditory cortex (AII) exhibited increased firing rates, while neuronal firing in AI and anterior auditory field (AAF) decreased (86). Moreover, elevated firing was detected in neurons tuned to higher frequencies, while at low frequency sites the opposite was true. These data implied that salicylate may exert effects via extra-lemniscal pathways, which provide an input for AII.

In the IC, the earliest evidence for salicylate-induced neural hyper-excitability came from Jastreboff and Sasaki (87), who measured spontaneous neuronal firing in the ICx of guinea pigs. Subsequently, bursting patterns of activity were discovered in the ICx in salicylate-administered rats (88), an effect most pronounced in neurons tuned to high frequencies. This is consistent with the finding that salicylate treatment often results in behavioral evidence of a high-frequency tinnitus percept (89). Changes in the IC following salicylate treatment are summarized in Table 3.

**Table 3:** **Pathophysiology in the IC associated with salicylate treatment**.

	**+**	−
**NEURAL ACTIVITY**		
Spontaneous activity		
Electro-physiology	Basta and Ernst (90), Jastreboff and Sasaki (87), and Manabe et al. (91)	Ma et al. (92)
Metabolic markers	Paul et al. (93)	Wallhausser-Franke et al. (94)
Burst firing	Chen and Jastreboff (88)	
Auditory-evoked activity	Ma et al. (92) and Sun et al. (95)	
**NEUROTRANSMISSION**		
Excitation
NMDA	Hu et al. (96) and Hwang et al. (97, 98)	
Inhibition
GABA_A_	Bauer et al. (99) (GABA_A_ affinity)	Bauer et al. (99) (GABA_A_ binding sites) and Zou and Shang (100)
GAD_65_	Bauer et al. (99)	
GAD_67_		Zou and Shang (100)
GlyR		Lu et al. (102)
Serotonin	Liu et al. (101)	Wang et al. (103)
**GENE EXPRESSION**		
Arg3.1/arc		Hu et al. (96)
Egr-1		Hu et al. (96)
c-FOS	Wu et al. (104)	Mahlke and Wallhausser-Franke (105)
**INFLAMMATORY MARKERS**		
COX-2		Hwang et al. (97)
IL1β	Hwang et al. (98, 106)	
TNFα	Hwang et al. (98, 106)	

*Elevations or enhancements in signaling are indicated by “+” and reductions by “−*.”

Increased excitability or enhanced metabolic activity in the IC were also demonstrated by others *in vivo* in guinea pigs (91), and in rats (93), as well as hyper-excitability in mouse brain slices (90). The latter study confirmed that salicylate acts at central targets, i.e., hyper-excitability does not occur simply as a result of peripheral effects. Indeed, in a later study, Basta et al. (107) showed that deafferented CN, MGB, and auditory cortex preparations were all susceptible to modulation of firing rates by salicylate, albeit with a lower sensitivity than the IC. The propensity for salicylate to directly alter central activity was further shown *in vivo* by Sun et al. (95). In their study, systemically administered salicylate increased the amplitude of sound-evoked field potentials in auditory cortex in awake rats, while *reduced* IC and cortical potentials were seen after direct administration of salicylate to the cochlea. Moreover, systemic administration also reduced the sound-evoked output of the cochlea, which – when considered alongside elevated cortical responses – suggests a change in central gain.

Contrastingly, Ma et al. (92) found a decrease in SFRs in the CNIC in mice following acute salicylate treatment. Moreover, this effect was strongest in neurons preferentially tuned to low frequencies. It should be noted that the salicylate dose administered by Ma et al. was somewhat lower than that used previously by others, although this was still sufficient to induce behavioral evidence of tinnitus in other rodents (108). Further evidence of decreased neural activity in the IC comes from a study in which salicylate treatment resulted in a reduction of 2-deoxyglucose activity in the IC, particularly in high-frequency regions (94).

Interestingly, Kumagai (109) demonstrated that salicylate-induced SFRs in AN fibers were only significantly elevated following administration of a high dose of salicylate, but not a lower dose. This provides a possible explanation for the disparity and heterogeneity between studies, with respect to IC hyper-excitability. However, given that relatively low doses result in behavioral evidence of tinnitus, it seems that increased excitation may be overly simplistic as a mechanism for salicylate-induced tinnitus.

A number of studies have examined the effects of salicylate on the balance of excitation and inhibition in the IC, in an attempt to understand the mechanisms underlying direct salicylate-induced hyper-excitability. Using the GPIAS approach to confirm tinnitus, Hu et al. (96) found that salicylate-induced reversible plastic changes in the IC. Specifically, they demonstrated an increase in the NR2B subunit of NMDA receptors, yet decreased expression of the immediate-early genes for Arc (activity-regulated cytoskeleton-associated protein) and Egr-1 (early growth response protein 1) in both the IC and auditory cortex of rats. The latter effect was somewhat surprising, given that both are normally associated with sensory-evoked neuronal activity. Salicylate-mediated changes in NR2B expression, as well as inflammatory mediators including tumor necrosis factor α (TNFα), cyclooxygenase-2 (COX-2), and interleukin-1β (IL1β), were also found in mice with behaviorally confirmed tinnitus (97, 98, 106).

Immediate-early gene expression was previously also examined in structures of the auditory and limbic systems of salicylate-treated gerbils (105). Although frequency-specific patterns of the arg3.1 gene (which translates to Arc) and c-Fos were apparent in auditory cortex, expression of these genes was limited in sub-cortical auditory structures, suggesting a lack of neuronal hyperactivity in the IC. Intriguingly, however, in the central nucleus of the amygdala both c-Fos and Arg3.1 were elevated following salicylate treatment, compared with saline-treated controls. Brainstem measurements of c-Fos from another study indicated an increase in the CNIC, while expression was negligible in the CN, in rats chronically dosed with salicylate (104), data which correlate with previous reports of salicylate-mediated increases in spontaneous activity in the IC.

With respect to components of inhibitory neurotransmission, early work involving chronic dosing of rats with salicylate demonstrated a number of changes to GABA signaling in the IC (99). Levels of GAD_65_ were elevated, while binding studies indicated increased GABA_A_ receptor affinity in CNIC, as well as in combined samples of ICx and ICd, which was coincident with a reduction in the number of GABA_A_ binding sites in CNIC. These effects correlated with behavioral evidence of salicylate-induced tinnitus in the same animals. This relationship between tinnitus behavior and changes in GABAergic signaling suggested that salicylate affected the balance of excitation and inhibition in the central auditory system, manifesting as a perceived tinnitus.

Contrastingly, Zou and Shang (100) found decreases in GABA_A_α1 and GAD_67_ expression in large GABAergic neurons in the IC 1 day after 5 days of chronic salicylate treatment, yet no change in levels of GAD_65_. This coincided with unchanged levels of the vesicular glutamate transporter VGLUT2, suggesting that glutamatergic input remained constant. It is worth highlighting that this study used a lower dose of salicylate than Bauer et al. (99) and measured levels of both GAD isoforms. Furthermore, Zou and Shang (100) concentrated on large GABAergic neurons in CNIC, thought to be the primary source of GABAergic input to the MGB from the IC. These factors do not necessarily explain the inconsistent results in GAD expression, but variability in methodology could underlie these differences.

Memantine, an NMDA receptor antagonist, did not abolish behavioral or neural evidence of tinnitus (108), which also suggests altered inhibitory drive in the midbrain is a contributing factor to tinnitus generation. In physiological terms, altered inhibitory neurotransmission may underlie enhancement of sound-evoked local field potentials (95) and broadening of excitatory receptive fields (92) in the IC. Others have shown the capacity for salicylate to interact with a number of neurotransmitter and neuromodulation systems. *In vitro* recordings from cultured rat IC neurons indicated non-competitive antagonism of α1 glycine receptor subunits (102). Furthermore, both *in vivo* and *in vitro* studies in rats point toward salicylate modulating serotonergic input (101, 103).

Salicylate-induced tinnitus is transient and hence studies that use salicylate are limited in translational value, in terms of providing signposts to understanding the chronic human condition, which is most often caused by noise exposure. However, salicylate remains a useful tool for making comparisons between a behavioral effect likened to tinnitus perception in humans, and potential underlying neural mechanisms. At this point in time – as discussed – the literature contains a variety of possible theories for salicylate-induced tinnitus, not least the effects this drug has in the IC. What seems clear, however, is that salicylate does induce central effects, and can directly affect both excitatory and inhibitory neurotransmission and plasticity in the IC. The effects of salicylate on the IC are summarized in Table 3. To generalize, studies suggest that salicylate (1) probably increases neural activity in the IC, (2) enhances excitation, (3) causes complex changes in inhibition, (4) reduces expression of immediate-early genes associated with neuronal activity (with one exception), and (5) initiates the production of some inflammatory mediators.

## Evidence from Tinnitus Patients That Implicates the IC

Collectively, data acquired using animal models of tinnitus implicate the IC in generating or maintaining the tinnitus percept. A degree of support for this idea is evident from work conducted with human subjects. Differences in the patterns of sound-evoked brain activation in the IC have been demonstrated in tinnitus patients with near-normal hearing (110), and in some instances, this was asymmetrical in patients with lateralized tinnitus, whereas bilateral tinnitus subjects exhibited symmetrical sound-evoked activation (111, 112). In a later study, Melcher et al. (113) suggest that asymmetrical activation coinciding with lateralized tinnitus actually constitutes a sub-group in terms of tinnitus classification. However, no discernible differences were apparent between controls and tinnitus patients when PET imaging was used to investigate previously reported hemispheric metabolic asymmetries in auditory cortex and the IC (114).

A reduction in functional connectivity between the IC and auditory cortices was also demonstrated in subjects with tinnitus, and interpreted as evidence to support failed thalamic gating in tinnitus patients (115), while no differences in either the magnitude or lateralization of functional MRI (fMRI) responses to auditory stimuli were seen in the IC. The idea of dysfunctional thalamic gating can be tentatively linked to the thalamocortical dysrhythmia model of tinnitus, proposed by Llinas et al. (116), which argues that disinhibition of auditory cortex as a consequence of abnormal thalamic input represents a putative mechanism for tinnitus. Indeed, using fMRI, patients with gaze-evoked tinnitus were found to exhibit less gaze-evoked inhibition of the auditory cortex, compared with controls (117). This was coupled with abnormal patterns of activation in the IC and inhibition in the MGB, and correlated with a perceived increase in tinnitus loudness. Elevations in IC neural activity measured directly in animals, and by more indirect means in humans, may be a prerequisite or a contributing component of the thalamocortical dysrhythmia model.

In addition to studies examining metabolic changes – suggestive of neural activity – a number of studies examining structural changes in the brain in tinnitus patients have identified altered morphology in the IC. Landgrebe et al. (118) identified a significant increase in gray matter in both the right IC and left hippocampus of their tinnitus group using structural MRI, compared with controls. This study originally aimed to replicate the findings of an earlier study (119), which showed subcallosal and thalamic volume changes; although regions of interest differed between studies, data from both suggest morphological volume changes in auditory and limbic brain areas relating to tinnitus pathophysiology, although others have failed to demonstrate volume changes in tinnitus patients [e.g., Ref. (120)]. White matter differences in the IC have also been examined in tinnitus patients, specifically comparing fiber tracts between IC and auditory cortex, IC and amygdala, and also between auditory cortex and the amygdala (121). Significant differences in this study were evident between the left IC and amygdala, right auditory cortex and IC, as well as bilateral auditory cortex and amygdala.

Several case reports also support a role for IC pathology in tinnitus. For example, Stimmer et al. (122) reported the sudden onset of a unilateral, right-sided tinnitus in a patient who exhibited prolonged auditory brainstem response (ABR) inter-peak latencies – which suggests the presence of pathology in the auditory pathway – and a small lesion in the left IC, presumed to have resulted from a transient, acute hemorrhage. Moreover, infarction in an area located near to the IC also coincided with a sudden worsening of reported tinnitus (123).

Changes in ABRs have been extensively studied and further implicate the IC and other brainstem structures in tinnitus pathology. Schaette and McAlpine (124) reported a significant reduction in wave I ABR amplitudes (generated by the auditory periphery), relative to the centrally generated wave V, thought to represent activity in the lateral lemniscus and IC. The authors proposed that this provided evidence for elevated central gain in the presence of reduced peripheral input. Subsequently, Gu et al. (125) also reported reduced wave I and augmented wave V amplitudes in tinnitus patients when compared with controls matched in age, sex, and hearing thresholds, and that ratio differences were most pronounced when comparing V:I and V:III (thought to represent activity in outputs from spherical bushy cells of the VCN). Interestingly, when these two groups were compared with a third cohort of younger, non-tinnitus subjects, elevated thresholds at mid-to-high frequencies were apparent in both groups, as was a reduction in wave I amplitudes, such that the observed peripheral dysfunction was not a unique indicator of tinnitus. Despite this, however, brainstem recordings performed in humans support a role for the IC and other brainstem structures in tinnitus pathology.

Somatosensory modulation of tinnitus is a well-established phenomenon (126). In animals, data suggest that the DCN is a key brainstem structure for the integration of somatosensory and auditory neural information that represents a neural correlate for such tinnitus modulation (127). Lanting et al. (128) examined neural correlates for somatosensory modulation of tinnitus by conducting fMRI experiments in patients capable of modulating tinnitus with jaw movements. Interestingly, this study found that jaw movements increased metabolic activity in both the CN and IC of tinnitus patients. Brain regions responsible for integrating somatosensory and auditory information, which includes the DCN and to a lesser extent the IC (129), were further implicated in tinnitus pathophysiology by a report from Gritsenko et al. (130), whereby lateralized tinnitus in an individual also exhibiting medial branch nerve degeneration was abolished by temporarily blocking C2–C3 nociceptive input.

To summarize, the majority of studies in humans have used either fMRI or structural MRI to examine the IC and have demonstrated the following: (1) changes in evoked activity in IC, (2) reductions in functional connectivity between IC and auditory cortex, (3) disparate morphological changes in the IC and other brain regions, and (4) altered brainstem responses implicating the IC. However, drawing firm conclusions regarding the role of the IC in the human condition is not feasible with the evidence available currently.

## Targeting IC Pathophysiology to Eliminate Tinnitus

There is currently no universally effective treatment for tinnitus. Consequently, a number of studies have addressed whether a range of interventions affect pathophysiological changes in the IC, caused by AOE. One approach has been to focus on GABA-enhancing drugs, aimed at restoring inhibition to suppress hyperactivity. Szczepaniak and Moller (131) demonstrated that l-baclofen, an antispasmodic GABA_B_ agonist, successfully attenuated hyper-excitability in the IC of AOE-treated rats. No behavioral testing was performed in this study to determine effectiveness on tinnitus. However, Zheng et al. (132) later demonstrated that a high dose of l-baclofen diminished tinnitus-like behavior in rats. While these animal data appear promising, the efficacy of this drug in eliminating tinnitus in humans is highly variable [for a review, see Ref. (133)].

Previous studies have demonstrated that tinnitus can persist following AN sectioning (6), which may even induce tinnitus in subjects that previously did not experience it (134, 135). In guinea pigs, Mulders and Robertson (7) demonstrated that, although IC hyperactivity could be reduced by cochlear ablation up to 6 weeks following AOE, there was no effect from 8 weeks onward (8). This suggests that central activity is dependent on peripheral drive in the early stages following AOE and later becomes centralized.

Recently, the loop diuretic furosemide was shown to reduce AOE-induced IC hyperactivity and behavioral evidence of tinnitus in guinea pigs within 6 weeks of acoustic trauma (136, 137). Putatively, this has been suggested to work via a reduction in the endolymphatic potential (138). Thus, it is likely to be most effective during the early stages of tinnitus development, when IC hyperactivity is dependent on cochlear input (7). Indeed, ~50% of patients experienced a reduction in tinnitus symptoms following intravenous administration of furosemide (139), an effect attributed to tinnitus being of cochlear origin. Paradoxically, however, high doses of furosemide actually appear to cause tinnitus in humans [see Ref. (140), for a review], so the efficacy for reducing tinnitus is as yet unclear.

An alternative and intriguing approach for modulating tinnitus-related pathology in the IC has recently been proposed as a result of work by Offutt et al. (141). These authors demonstrated, in guinea pigs, that electrical stimulation of the ICd resulted in either suppression or facilitation of firing rates in the CNIC, and postulated that a midbrain implant could be used to reduce or even eliminate tinnitus. As yet, these effects have not been demonstrated in AOE-treated animals, or indeed in animals displaying evidence of tinnitus, so the viability of this intervention remains to be determined.

Tinnitus can briefly be reduced or eliminated following the presentation of a masking sound stimulus, a phenomenon referred to as residual inhibition (142). Voytenko and Galazyuk (143) suggested that suppression of activity in awake mouse IC neurons by a preceding sound stimulus represents a possible underlying mechanism for residual inhibition. While this intervention only produces a temporary cessation of tinnitus, it nonetheless provides a useful tool for comparing neural pathology underlying tinnitus to a brain state wherein tinnitus is absent.

To date, intervention-centered research has been hampered by a lack of differentiation between underlying tinnitus pathology and effects that simply relate to AOE. Without this, the efficacy of treatment approaches is difficult to appraise.

## The IC as a Component in Putative Models of Tinnitus

The central gain hypothesis proposes a reduction in cochlear output concurrent with a paradoxical sustained enhancement of central activity [for a recent review, see Ref. (144)]. This likely reflects homeostatic mechanisms initiated to sustain the mean level of firing within the auditory brain (145, 146). Increases in the steepness of local field potential amplitude-sound level functions, despite a loss of peripheral sensitivity, implied that this central enhancement was evident at the level of the IC (21, 147).

While the central gain hypothesis is persuasive, and is perhaps most pertinent when considering IC involvement, it is highly likely that tinnitus perception involves complex interactions with other brain areas (148). Given the strong emotional aspects of tinnitus, it has previously been suggested that input from limbic areas (149), specifically to the MGB (150), likely underlie the awareness of tinnitus. Connections between the MGB and limbic areas are prevalent, and presumably are involved in modulating responses to auditory stimuli (150). Moreover, there are also direct connections between the CNIC and the amygdala (151). However, it is as yet unclear whether limbic-auditory interactions at the level of the IC are altered in a way that correlates with tinnitus. Examining limbic-auditory interactions is currently *en vogue* in tinnitus research, although clearly this is a challenging question to pose in animal models. Nevertheless, limbic-auditory interactions remain an intriguing avenue for inquiry. In particular, pathological interactions could underlie differences in tinnitus susceptibility, since some people with peripheral damage do not develop tinnitus, while others do (152).

A large proportion of patients demonstrate tinnitus modulation by jaw movements or neck muscle contractions (153, 154). This phenomenon is likely the result of interactions between auditory and somatosensory neural circuitry [for a review, see Ref. (155)]. It has previously been shown that stimulation of somatosensory areas modulated firing in cochlear nucleus neurons (156–158), and that this modulation was altered in the presence of behavioral evidence of tinnitus (127). There are also direct and prominent connections to the ICx from somatosensory areas (159, 160), and IC activity was modulated by somatosensory stimulation (161). Thus, it would be of considerable interest to determine whether changes in IC-somatosensory system interactions correlate with behavioral evidence of tinnitus; this may also further elucidate the mechanisms underlying somatosensory modulation of tinnitus.

## Conclusion

Studies using animal models imply that the IC plays an important role in tinnitus pathology. This is the case regardless of whether tinnitus is induced by AOE or salicylate. Animal models allow for invasive studies, but carry with them the fundamental difficulty of establishing whether animals actually perceive tinnitus. The introduction of behavioral tests attempting to identify tinnitus allows researchers to correlate neural changes with the presence of a tinnitus percept, although existing tests have their caveats. Human studies are important for directly relating changes in the brain to the human condition, although generally do not allow for invasive recording techniques and current measures are limited in their spatial resolution (e.g., EEG) or temporal resolution (e.g., fMRI or PET). Currently, there are clear disparities in the literature that need to be resolved, namely, clarifying the time-course of changes post-AOE, and separating tinnitus-related effects from those attributable simply to noise exposure. There is also a lack of clarity in the contributions of different IC sub-divisions to tinnitus pathophysiology. Elucidating these characteristics of tinnitus pathology will be undeniably difficult, but will likely prove essential to facilitate development of a treatment to eliminate the tinnitus percept.

## Conflict of Interest Statement

The authors declare that the research was conducted in the absence of any commercial or financial relationships that could be construed as a potential conflict of interest.
